# 2019 FDA TIDES (Peptides and Oligonucleotides) Harvest

**DOI:** 10.3390/ph13030040

**Published:** 2020-03-05

**Authors:** Danah Al Shaer, Othman Al Musaimi, Fernando Albericio, Beatriz G. de la Torre

**Affiliations:** 1 KRISP, School of Laboratory of Medicine and Medical Science, College of Health Sciences, University of KwaZulu-Natal, Durban 4001, South Africa; 217078895@stuukznac.onmicrosoft.com (D.A.S.); 217078894@stuukznac.onmicrosoft.com (O.A.M.); 2 School of Chemistry and Physics, University of KwaZulu-Natal, Durban 4001, South Africa; 3 CIBER-BBN, Networking Centre on Bioengineering, Biomaterials and Nanomedicine and Department of Organic Chemistry, University of Barcelona, 08028 Barcelona, Spain

**Keywords:** afamelanotide, bremelanotide, DOTATOC, drugs, ^68^Ga-DOTATOC, enfortumab vedotin, golodirsen, givosiran, polatuzumab vedotin, oligonucleotides, peptides, pharmaceutical market

## Abstract

2019 has been an excellent year in terms of peptides and oligonucleotides (TIDES) approved by the FDA. Despite the drop in the number of total drugs approved by the FDA in 2019 in comparison with 2018 (48 vs. 59), the total number of TIDES authorized increased (seven vs. three). Year after year, TIDES are increasingly present in therapy, as imaging agents, theragnostic and constituent moieties of other complex drugs, such as antibody drug conjugates. This means a consolidation of these kinds of drugs in the pharmaceutical arena, paving the way in the coming years for the approval of others for diverse medical indications. Here the TIDES approved in 2019 are analyzed in terms of chemical structure, medical target, mode of action, and adverse effects.

## 1. Introduction

**Figure 1:**
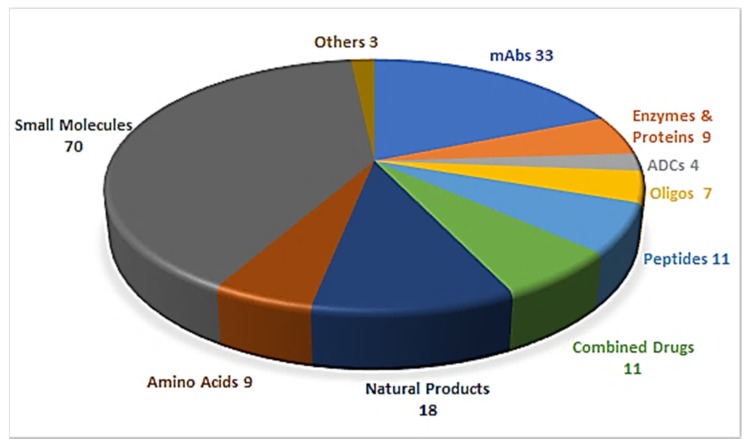
A total of 175 new drugs approved by the FDA from 2016 to 2019 [1,2,3,4]. mAbs; Monoclonal antibodies, ADCs; antibody drug conjugates, Oligos; oligonucleotides.

Drug discovery is a multifactorial activity involving the private and public sectors and, more importantly, society as a whole, which is represented by patients. From 2016 to 2019, the United States Food and Drug Administration (FDA) approved a total of 175 new drugs for commercialization (Figure 1) [1,2,3,4]. Forty-eight drugs were approved in 2019 [4], 10 of which were biologics and the remaining 38 new chemical entities (NCEs). The peptides and oligonucleotides (TIDES) class is manufactured chemically and thus belongs to NCEs; however, these molecules have a clear biological structure and could; therefore, be considered a transition between the two subclasses. In 2019, five TIDES (three peptides and two oligonucleotides) were authorized (Table 1). This figure accounts for approximately 10% of the total drugs approved and agrees with the total number approved in the period 2016–2019 (18 TIDES: 11 peptides and seven oligonucleotides, vs. 175). Furthermore, three antibody drug conjugates (ADCs) were approved. In two of these, namely enfortumab vedotin-ejfv and polatuzumab vedotin-piiq, the payload is the peptide monomethyl auristatin E (MMAE), derived from the marine mollusk dolastatin. Finally, peptides serve as linkers between the payload and the antibody in all three ADCs. Thus, TIDES are present in eight of the 48 drugs approved in 2019. These figures reflect the high relevance of such molecules for biomedical applications.

Here, TIDES from the 2019 harvest are discussed from a molecular perspective, application as a drug, mode of action, and adverse effects.

## 2. Oligonucleotides

### 2.1. Golodirsen (Vyondys 53^TM^)

**Figure 2:**
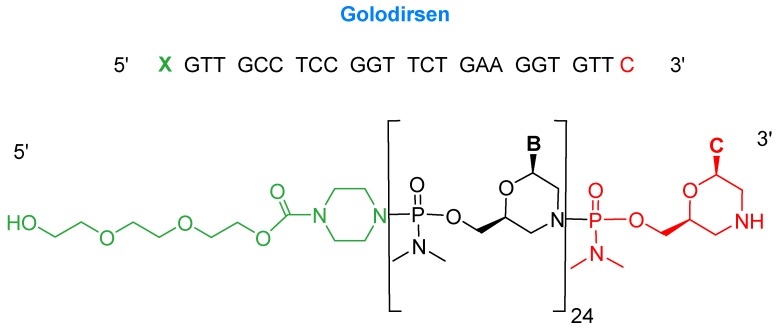
Chemical structure of golodersin (Vyondys 53^TM^).

Golodirsen is an antisense oligonucleotide with a single strand of 25 monomers [6]. The subunits are linked through a synthetic neutral phosphorodiamidate morpholino oligomer (PMO) backbone. This neutral backbone confers greater stability to the strand than the natural negatively-charged phosphodiester linkage. The nitrogenous bases are incorporated on a morpholine six-membered heterocycle instead of the natural five-membered ribose (or deoxyribose) ring. The strand ends with a small hydrophilic triethylene glycol chain. Golodirsen has a molecular weight of 8647.4 Da (Figure 2).

Golodirsen was developed for the treatment of Duchenne’s muscular dystrophy (DMD), which is a progressive muscle deterioration that starts in early childhood and in most cases ends up crippling patients before adolescence. After several years, patients die mainly from heart failure [7,8]. This disorder is caused by a deletion mutation in the dystrophin gene, which transcribes for the production of dystrophin, a huge protein that covers muscular fibers, protecting them from damage upon contraction and enhancing muscle performance. The genetic disorder causes the production of a non-functioning dystrophin protein and consequently muscle wasting. This gene is linked to the X chromosome, thus making DMD disorder more noticeable in male infants [7,9].

The dystrophin gene consists of 79 exons. Some genetic mutations cause the deletion of exon 52, which blocks the translation process [7]. Golodersin conceals exon 53, allowing translation to take place and producing a protein with some missing parts but still functional [9,10]. A similar previous drug, eteplirsen (Exondys 51), was the first FDA-approved antisense (in 2016) therapy for the treatment of the same disorder by exon 51 skipping [10].

Golodersin is administered intravenously [10]. Some adverse effects include headache, pyrexia, fall, abdominal pain, nasopharyngitis, cough, vomiting, and nausea [11].

Both golodirsen and eteplirsen were developed by Sarepta Therapeutics. Golodersin was approved by the FDA on 12 December 2019 [12], and there are some concerns about its renal toxicity [10,11].

### 2.2. Givosiran (Givlaari^TM^)

**Figure 3:**
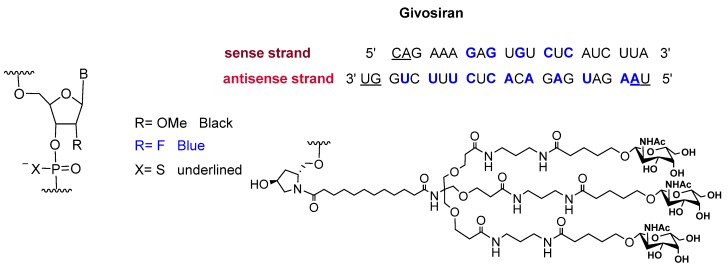
Chemical structure of givosiran (Givlaari^TM^) [13].

Givosiran is the second small interfering RNA (siRNA) drug to be approved by FDA [13] (the first one was patisiran (Onpattro^TM^), which was authorized in 2018 for the treatment of hereditary transthyretin-mediated amyloidosis and targets hepatic cells [14,15]). Additionally, givosiran is the first approved drug that demonstrates the enhanced stabilization chemistry (ESC)-GalNAc-siRNA conjugate technology. This technology involves several synthetic RNA stabilization chemistries. The 2′-OH of the ribose in some monomers are methylated (forming 2′-O-methyl-ribonucleoside), while in others they are substituted by the highly electronegative fluorine atom (2′-F-ribonucleoside) in order to boost the stability of the double strands against the nuclease [16]. In addition, the hepatocyte-targeting ligand that is attached to the 3′ terminal of the sense strand has three N-acetylgalactosamine moieties. The other three terminals (5′ of the sense strand, and 3′,5′ of the antisense strand) have thiophosphate linkages in the last two subunits for each side. This conjugation (ESC-GalNAc-siRNA) confers enhanced stability upon subcutaneous administration of the siRNA and offers a 10-fold increased potency of the drug over the standard template chemistry (STC) [13,17]. Givosiran is prepared as the sodium salt of a double-strand oligonucleotide (sense and antisense strands) (Figure 3) and it has a molecular weight of 17,245.56 Da. 

Givosiran was developed for the treatment of acute hepatic porphyria in adults, which is a genetic disorder that results in the accumulation of the neurotoxic intermediates aminolevulinic acid (ALA) and porphobilinogen (PBG) during the hemes production cycle (the hemoglobin oxygen binding site) in hepatic cells. This disorder causes severe abdominal pain, nausea, vomiting, and constipation and it can be triggered by several factors, such as certain drugs, low sugar intake due to fasting, smoking, and stress [18,19].

The N-acetylgalactosamine ligand bound to the sense strand facilitates the uptake into liver cells [19]. After entering hepatocytes, it binds to and silences aminolevulinate synthase 1 (ALAS1) mRNA, thereby halting ALA production and consequently preventing the accumulation of the toxic intermediates in body tissues [18].

Givosiran is administered subcutaneously and is well-tolerated. However, regular check-ups for liver and kidney function are highly recommended [19]. It was developed by Alnylam Pharmaceuticals Inc. (Cambridge, Massachusetts, United States) (the same company that developed patisiran) and approved by the FDA on 20 November 2019 [20].

## 3. Peptide-Based Drugs

### 3.1. [^68^Ga]Ga-DOTATOC ([[^68^Ga]Ga-DOTA, Tyr3]-Octreotide)

**Figure 4:**
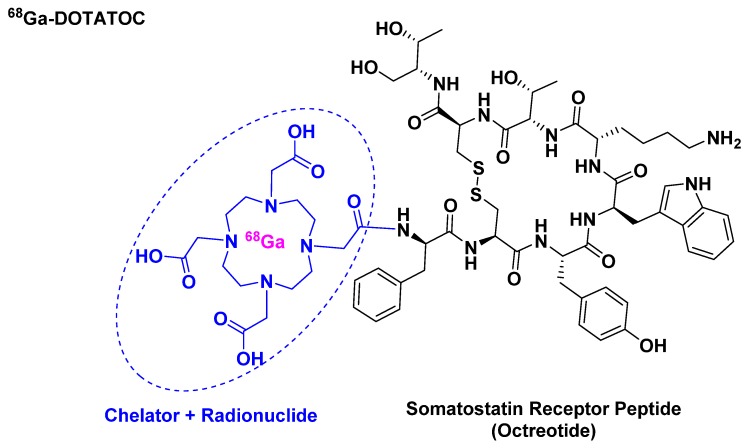
Chemical structure of ^68^Ga-DOTATOC.

^68^Ga-DOTATOC belongs to the peptide receptor radionuclide therapy (PRRT) class and it is used for scintigraphic imaging [21]. It is composed of ^68^Ga, which has a half-life of 68 min and a high positron abundance [22,23], as radionuclide, which is chelated by DOTA that in turn is attached to [Tyr^3^]-octreotide (Figure 4). It is used mainly in positron imaging therapy (PET) for the detection of somatostatin receptor-positive neuroendocrine tumors (NETs).

^68^Ga-DOTATOC is related to ^68^Ga-DOTATATE, which was approved in 2016. Both share almost the same affinity towards the somatostatin receptor (sstr). The in vitro affinity of ^68^Ga-DOTATATE towards sstr subtype 2 (sstr2) is about 10-fold that of ^68^Ga-DOTATOC [24]. Nevertheless, ^68^Ga-DOTATOC is able to detect more NET lesions than the DOTATATE analogue with highly reproducible imaging efficiency [22]. Furthermore, the higher in vitro affinity of the DOTATATE analogue has not proven to be relevant clinically. These studies showed that ^68^Ga-DOTATOC has a higher affinity towards sstr2 receptor than the ^68^Ga-DOTATATE as demonstrated by higher tumor uptake values. This could explain the superiority of ^68^Ga-DOTATOC for more lesion detection [24].

A study by L. K. Khor et al. has shown that ^68^Ga-DOTATOC is a good biomarker for newly-diagnosed undifferentiated nasopharyngeal carcinomas (NPCs), and to a lesser extent for recurrent NPC and metastatic nodes [25].

Prior to ^68^Ga-DOTATOC, ^111^In-octreotide was the only FDA-approved imaging agent for somatostatin receptor scintigraphy of NETs [22]. ^111^In-octreotide utilizes the single-photon emission computed tomography (SPECT) technique.

Using [^111^In]In-octreotide for imaging of NETs results in a higher dose to the patient (12 mSv) as compared to the use of [^68^Ga]Ga-DOTATOC (4.26 mSv) [22]. This is due to the longer half-life of ^111^In (T1/2 = 2.8 d) as compared to ^68^Ga (T1/2 = 68 min).

^68^Ga-DOTATOC is administered intravenously and has some adverse effects, including nausea, pruritis, and flushing [26]. It was developed by the University of Iowa Health Care (UIHC) and approved by the FDA on 21 August 2019 [27]. From the same family, in 2018 the FDA approved [^177^Lu]Lu-DOTA-TATE ([[^177^Lu]Lu-DOTA0, Tyr3]-octreotate) for the treatment of gastroenteropancreatic neuroendocrine tumors (GEP-NETs) [28].

### 3.2. Afamelanotide (Scenesse^®^)

**Figure 5:**
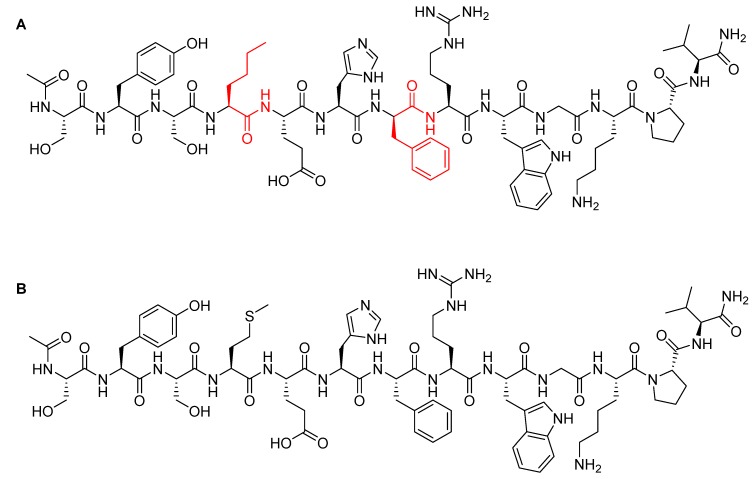
Chemical structure of: (**A**) Afamelanotide (Scenesse^TM^); (**B**) α-melanocyte stimulating hormone (α-MSH). Differences are shown in red.

Afamelanotide is a synthetic tridecapeptide structural analogue of α-melanocyte stimulating hormone (α-MSH) with a molecular weight of 1646.87 Da (Figure 5A) [29]. It differs from the natural analogue (Figure 5B) in its fourth and seventh amino acid residues, in which Met and Phe are replaced by norleucine (Nle) and D-Phe, respectively [30]. Such modifications play a key role in enhancing the properties of afamelanotide versus the physiological α-MSH analogue. In this regard, afamelanotide shows enhanced resistance against enzymatic degradation, increases biological activity, and prolongs the plasma half-life [30] by stimulating binding affinity with melanocortin 1 receptor (MC1R) [31]. Furthermore, unlike other small therapeutic peptides, afamelanotide has a minimal risk of inducing anti-drug antibodies even after six years of continuous treatment [31,32]. In contrast, anti-drug antibody induction has been reported for its α-MSH natural analogue, in which a noticeable increase of IgM autoantibodies against α-MSH was observed [33].

Afamelanotide is used for the treatment of erythropoietic protoporphyria (EPP) [29]. It provides photoprotection upon exposure to direct sunlight by increasing the density of eumelanin in the skin—this is called the skin tanning process [34]. It works like α-MSH, binding to the G-protein-coupled MC1R in dermal cells and stimulating the production of melanin, along with consecutive biological processes [35]. Despite stimulating melanin synthesis in the same way, afamelanotide is considered a preventive therapy. In which, unlike α-MSH natural analogue, afamelanotide induces eumelanin synthesis in advance and independently of having UV-damaged skin cells [32].

Afamelanotide is administered subcutaneously [36] through a poly(lactic-co-glycolic acid) (PLGA) biodegradable polymer [34]. It has a half-life of 30 to 50 min, after which it is hydrolyzed into shorter peptides and amino acid residues [30,32]. Most of the active materials are excreted within two days, and by 10 days its plasma levels are below the limit of quantification [30,32].

Afamelanotide has some adverse effects, including, but not limited to, nausea, vomiting, flushing, headache, cough, fatigue, and dizziness [36].

Its development was started in the 1980s by Tomi Sawyer and Victor Hruby at the University of Arizona [29], then Clinuvel Inc. performed the required clinical studies and brought the product to the market. It has been sold in Switzerland and Italy since 2013 [35], but was only approved by FDA on 8 October 2019 [37].

### 3.3. Bremelanotide (VYLEESI^TM^)

**Figure 6:**
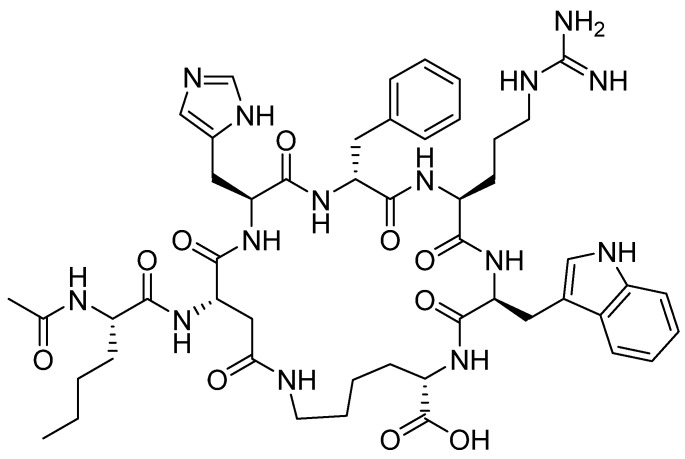
Chemical structure of bremelanotide (Vyleesi^TM^).

Bremelanotide is a homodetic side-chain to tail cyclic heptapeptide with the sequence of Ac-Nle-*cyclo*[Asp-His-DPhe-Arg-Trp-Lys]-OH [38]. The cycle through an amide bond is between the ß carboxylic acid of Asp ε amino of the Lys, which is the C-terminal residue. The exocyclic Nle is acetylated. It has a molecular weight of 1025.182 Da. The structure is shown in Figure 6.

Bremelanotide is an analogue of the natural α-MSH [39]. It works as an agonist of melanocortin receptors and is used to treat hypoactive sexual desire (HSDD) in women of fertile age. This disorder results in a low sex drive that is not caused by other factors such as medications or any medical or psychiatric condition [40,41].

The drug is administered subcutaneously and possible adverse effects include nausea, flushing, injection site reactions, headache, and vomiting [42,43].

Bremelanotide was first studied by Arizona Cancer Research Centre as a self-tanning inducer. However, increased sexual desire in patients was observed as a side effect [44]. Later on, it was developed by Palatin technology as a treatment for HSDD [44] and then out-licensed to AMAG PHARMS and approved by the FDA on 21 June 2019 [45].

Although bremelanotide and afamelanotide belong to the same α-MSH hormone-analogous family, their structures show some differences. Bremelanotide resembles the middle section of afamelanotide with the absence of the first three residues Ser-Tyr-Ser at the N-terminus and the last two residues Pro-Val at the C-terminus, in addition to the Gly, the tenth residue. Additionally, bremelanotide comprises a side-to-side amide bond that forms the cycle.

## 4. Peptides as Payloads in ADCs

### 4.1. Enfortumab Vedotin-Ejfv (PADCEV^TM^)

**Figure 7:**
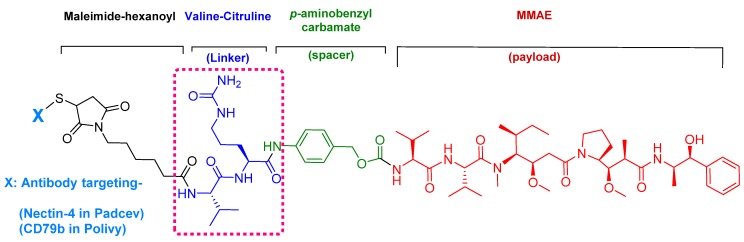
Chemical structure of enfortumab vedotin-ejfv (Padcev^TM^) and polatuzumab vedotin-piiq (Polivy^TM^). MMAE; monomethyl auristatin E.

Enfortumab vedotin-ejfv is an ADC therapy [46], which is an emerging therapeutic strategy for transporting cytotoxic chemotherapeutic agents to certain tumors [47]. This drug has three main components: payload/cytotoxic drug, mAb, and linker [47]. The antibody comprises human monoclonal antibody (enfortumab) and targets nectin-4 (also known as poliovirus receptor-related protein 4 (PVLR4)), which is highly expressed in NETs [46]. Enfortumab is derived either from a Chinese hamster cell ovary line [ASG-22CE] or can be prepared via murine hybridoma technology (AGS-22M6E (or ASG-22ME)) [48]. Enfortumab is conjugated to MMAE (vedotin) via a cathepsin-cleavable linker (Figure 7) [46].

**Figure 8:**
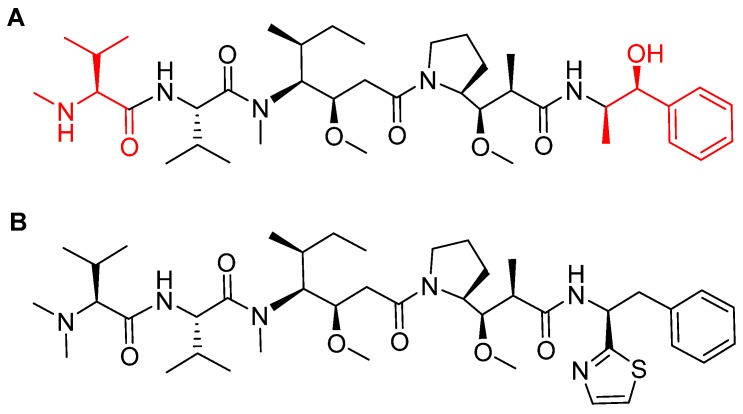
Chemical structure of: (**A**) Synthetic monomethyl auristatin E (MMAE) analogue; (**B**) natural dolastatin 10. Differences are shown in red [50].

MMAE, the cytotoxic component [49], is a synthetic pentapeptide (717.99 Da) that works as a potent microtubule-disrupting agent [48]. It is a structurally-modified analogue of the natural dolastatin 10 [50], a potent antineoplastic pentapeptide (785.1 Da) isolated from the marine mollusk *dolabella auricularia* by Pettit et al. in 1987 [51]. MMAE comprises the following four amino acid residues: dolavaline (Dov), Val, dolaisoleuine (Dil), dolaproine (Dap), and the C-terminal amine dolaphenine (Doe) [52]. Figure 8 shows the structural differences between the synthetic MMAE analogue (A) and the natural pentapeptide dolastatin 10 (B).

Given that MMAE is a peptide, it is metabolized into smaller non-toxic amino acid fragments and then recycled or excreted by the body [53].

Nectin-4 is a 66 KDa protein that is expressed in several cancer tissues (breast, lung, bladder, among others) and highly expressed in urothelial cancer [54]. Among other nectins, it is considered a potential target due to its distinguished sequence in its family (1, 2, or 3) with low degree of similarity with other family members [55]. Furthermore, the limited expression of nectin-4 in normal tissues minimizes the possibility of these tissues being targeted during the course of the treatment [46].

Enfortumab vedotin-ejfv is a pan-fibroblast growth factor receptor (FGFR) suppressor [56]. It is indicated for the treatment of adult patients with locally advanced or metastatic urothelial cancer who previously received immune checkpoint therapy [programmed death receptor-1 (PD-1) or programmed death-ligand 1 (PD-L1) inhibitor] [56]. After platinum-based chemotherapy, enfortumab vedotin-ejfv is prescribed as a second line treatment for patients with susceptible FGFR2 or FGFR3 [48,56].

The drug binds to nectin-4-expressing cells. The resulting complex is internalized into the cell and then the valine-citruline (Val-Cit) dipeptide linker is recognized and cleaved by cathepsin-B in the tumor cell [57]. Consequently, the cytotoxic MMAE is selectively released, thereby leading to apoptosis [46].

It is administered intravenously. Common adverse effects include fatigue, peripheral neuropathy, decreased appetite, rash, alopecia, nausea, dysgeusia, diarrhea, dry eye, pruritus, and dry skin [58].

It was developed by Astellas Pharma and granted accelerated approval by the FDA on 18 December 2019 [59].

### 4.2. Polatuzumab Vedotin-Piiq (Polivy^TM^)

**Figure 9:**
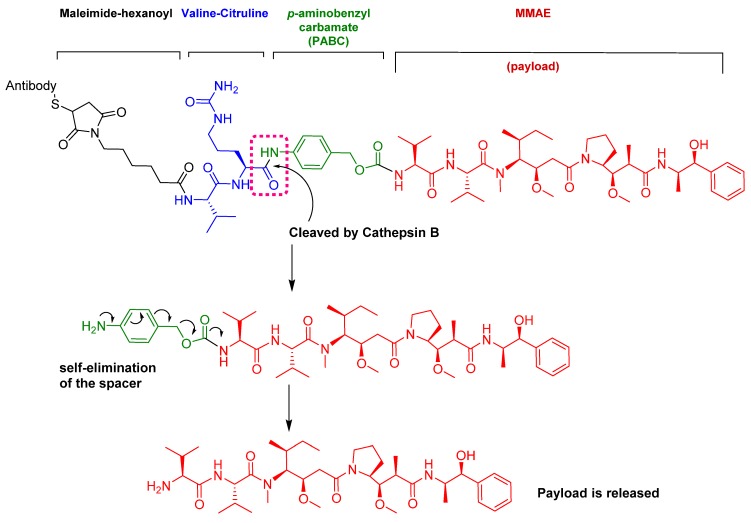
Mechanism of payload release in ADCs with Val-Cit linker and p-aminobenzyl carbamate as a spacer [67]. MMAE; monomethyl auristatin E.

Polatuzumab vedotin-piiq is an ADC therapy [60]. It comprises the same linker and payload as in the previous drug (enfortumab vedotin-ejfv), but a different antibody (Figure 9). It is prescribed as a combination with bendamustine and rituximab (BR combination) [5] and is used for the treatment of adults with relapsed or refractory diffuse large B-cell lymphoma [61].

It selectively binds to CD79b that is overexpressed in mature B-cells [61]. Following the same mechanism in enfortumab vedotin-ejfv which ends by cell apoptosis.

Of note, the tolerability and safety profile of this drug was accepted for non-Hodgkin’s lymphoma (NHL) patients but not for those with chronic lymphocytic leukemia (CLL) [61].

It is administered intravenously. Common adverse effects include neutropenia, thrombocytopenia, anemia, peripheral neuropathy, fatigue, diarrhea, pyrexia, decreased appetite, and pneumonia [62].

It was developed by Roche and granted accelerated approval by the FDA on 10 June 2019 [63].

## 5. Peptides as Linkers in ADCs

### 5.1. Val-Cit

The choice of a suitable linker is a highly sensitive step in ADC manufacturing. First, the conjugate should be stable enough during its circulation in blood serum to avoid damaging body tissues. Second, the programable release of cargo should be easily triggered once the conjugate reaches its target. Thus, a suitable linker should successfully combine serum stability and in-target lability without adversely affecting the stability of the antibody itself upon conjugation [64,65].

Among the four known types of linkers, namely hydrazones, disulfides, peptides, and thioethers [57], short peptidyl linkers, such as Val-Cit dipeptide, fulfill the requirements for this critical function and even outperform the tetra-peptidyl linkers (Gly-Phe-Leu-Gly and Ala-Leu-Ala-Leu) previously used and that showed some aggregation issues upon conjugation [57,64,65]. The premature release of the payload in the case of hydrazone (due to pH changes) and disulfide linkers (due to exchange with other thiols, such as glutathione) may influence the potency of the treatment, while the delayed release of cargo in the case of thioether linkers (payload is released only after total degradation of the antibody) may cause the loss of anticancer activity [65,66]. In Padcev^TM^ and Polivy^TM^, a maleimidocarpoyl moiety is added to the N-terminal of the dipeptide Val-Cit to facilitate conjugation to the antibody. The amide bond formed by the C-carboxyl group of the Cit, which is linked to a self-immolative p-amino benzyl carbamate (PABC) spacer, is stable in serum and can be rapidly hydrolyzed by lysosomal cathepsin-B releasing the PABC-MMAE moiety. This undergoes self-elimination liberating the payload MMAE [57]. The mechanism of drug release is shown in Figure 9.

### 5.2. Gly-Gly-Phe-Gly

The ADC fam-trastuzumab deruxtecan-nxki (Enhertu^TM^) targets human epidermal growth factor receptor-2 (HER2) [66]. It is used for the treatment of adult patients with unresectable or metastatic HER2-positive breast cancer [68].

**Figure 10:**
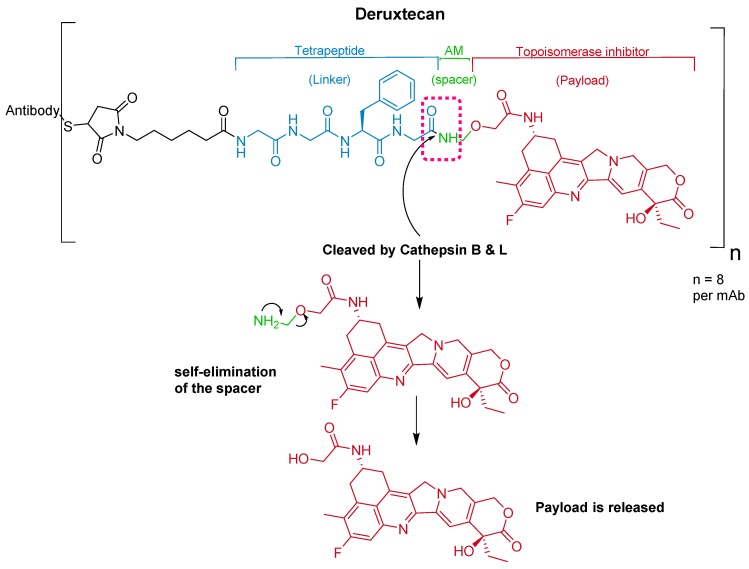
Chemical structure of fam-trastuzumab deruxtecan-nxki (Enhertu^TM^) showing the tetrapeptide linker and amino methylene cleavage in the payload-release process. AM; aminomethyl.

Trastuzumab is conjugated to the antibody via a tetrapeptide linker, Gly-Gly-Phe-Gly (Figure 10), which is cleavable by lysozymes [69]. The linker is connected to a Cys residue of the mAb through a maleimidocarpoyl component [66] and to a self-immolative amino methylene (AM) spacer [70].

It was developed by Daiichi Sankyo and approved by the FDA on 20 December 2019 [71].

## 6. Conclusions

The 2019 year has been very successful regarding the role of TIDES in the drug arena. Thus, in addition to approving two oligonucleotides and three peptide APIs, the FDA authorized two ADCs with a peptide as payload. Finally, three ADCs (including the former two) have a peptide-based linker. Therefore, eight of the 48 drugs (more than 15%) approved this year were or contained TIDES. While the peptide market is already consolidated through the large number of authorized peptides, the seven oligonucleotides recently (2017–2019) given the green light pave the way for the approval of others for diverse medical indications. Of note, is the presence of peptides as payloads and as a part of the linkers in the ADCs.

## Figures and Tables

**Table 1:** Summary of the 2019 FDA peptides & oligonucleotides (TIDES) harvest [5].

#	Active Ingredient Trade Name	Type	Indication	Target	Route
**1**	Golodirsen Vyondys 53^TM^	Antisense oligonucleotide	Duchenne’s Muscular Dystrophy (DMD)	Exon 53 in dystrophin gene	Intravenous
**2**	Givosiran Givlaari^TM^	Antisense oligonucleotide	Acute Hepatic Porphyria (AHP)	Aminolevulinate synthase 1 (ALAS1) mRNA	Subcutaneous
**3**	^68^Ga-DOTATOC	Peptide	Scintigraphic imaging	Somatostatin receptor	Intravenous
**4**	Afamelanotide Scenesse^TM^	Peptide	Erythropoietic protoporphyria (EPP)	Melanocyte-stimulating hormone receptor	Subcutaneous
**5**	Bremelanotide Vyleesi^TM^	Peptide	Hypoactive sexual desire disorder	Melanocyte-stimulating hormone receptor	Subcutaneous
**6**	Enfortumab vedotin-ejfv Padcev^TM^	ADC with peptide payload and linker	Urothelial cancers	Nectin-4 receptor	Intravenous
**7**	Polatuzumab vedotin-piiq Polivy^TM^	ADC with peptide payload and linker	Refractory diffuse large B-cell lymphoma	CD79b receptor expressed in mature B-cells	Intravenous
**8**	Fam-trastuzumab deruxtecan-nxki Enhertu^TM^	ADC with a peptide linker	Unresectable or metastatic HER2-positive breast cancer	Human epidermal growth factor receptor-2 (HER2)	Intravenous

ADC; antibody drug conjugate.
